# The potential role of major dissection observed on optical frequency-domain imaging in optimal lumen expansion after coronary intravascular lithotripsy: a comparative analysis with calcium fractures

**DOI:** 10.1007/s12928-025-01145-2

**Published:** 2025-06-11

**Authors:** Kotaro Miyata, Taku Asano, Takahiro Suzuki, Masafumi Ono, Jiro Aoki

**Affiliations:** Department of Cardiovascular Medicine, St. Luke’s International Hospital, St. Luke’s International University, 9-1 Akashi-Cho, P. O. Box 104-8560, Chuo-Ku, Tokyo Japan

Intravascular lithotripsy (IVL) is a novel device for preparing severely calcified lesions. Its acoustic pressure waves induce calcium fractures, reducing the caging force and enabling larger stent expansion [1]. IVL also frequently causes major dissections, such as luminal tissue separation between calcium and adjacent plaque layers (Fig. 1A) [2]. These dissections, driven by balloon expansion and pressure waves, may further reduce the caging force by separating calcium from the vessel wall. However, their impact on stent expansion remains unclear.

**Fig. 1:**
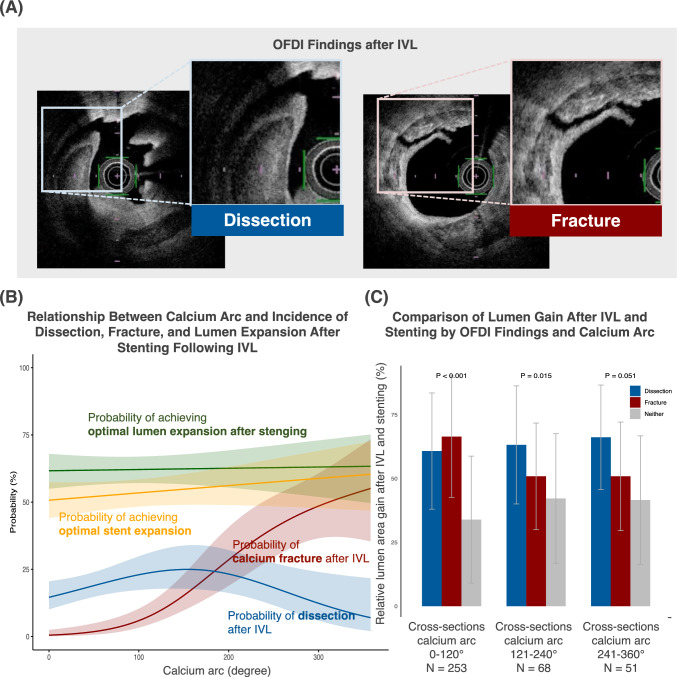
The incidence of major dissection and fractures after intravascular lithotripsy and their impact on optimal stent expansion. *OFDI* optical frequency-domain imaging, *IVL* intravascular lithotripsy

Our single-centre study analyzed 12 patients undergoing optical frequency-domain imaging (OFDI)-guided PCI with IVL for severely calcified lesions. Balloon and stent sizes were determined at the operator’s discretion based on OFDI measurements acquired prior to each procedural step. For the analysis, serial OFDI images were obtained pre-IVL, post-IVL, and post-stent. Cross-sectional images, analyzed at 1 mm intervals using QIvus software (Medis Medical Imaging, Leiden, The Netherlands), were matched across each time point using anatomical landmarks. Baseline analyses included calcium arc and maximum thickness. Post-IVL, fractures (defined as visible breaks in previously continuous calcium) and major dissections (luminal tissue separation extending into deeper layers [media or adventitia] or occurring between calcified plaque and adjacent tissue layers) were assessed. Eccentricity index (EI) was calculated as the ratio of the minimum and maximum luminal diameter per cross-section. Relative lumen area gain was calculated as post-stent lumen area gain divided by the baseline reference lumen area (the average of the mean lumen areas at the 5 mm proximal and distal edge segments).

A total of 372 matched OFDI cross-sections from 12 lesions were analyzed. Patients had a median age of 75.9 years (41.7% female), and stents averaged 3.09 ± 0.40 mm in diameter. The mean OFDI calcium score was 3.73 ± 0.45 [3]. Among 12 cases, pre-IVL predilatation was performed in 33.3% using semi-compliant, scoring, or cutting balloons. IVL was conducted with an average balloon size of 2.68 mm, pressures of 4 atm (during pulsation) and 6 atm (post-pulsation), and 74.2 pulses. Post-IVL predilatation was rare (16.7%) and used only non-compliant balloons. DES implantation was performed in all cases (average size 3.09 mm), and postdilatation was done in 58.3% using non-compliant balloons (3.67 mm). Post-IVL, major dissections were observed in 17.7% and fractures in 11.6%. Probability curves derived from logistic regression models using a natural spline (Fig. 1B) showed major dissection incidence peaking at moderate calcium arcs (100–220°) and fracture probability rising sharply beyond 100°. Despite calcium severity, optimal lumen/stent expansion, defined as a post-stent lumen/stent area exceeding the baseline reference area, remained consistently high. Figure 1C illustrates the relative lumen area gain after IVL and stenting across calcium arc groups. Both major dissections and fractures contributed to lumen gain. The EIs after stent implantation were comparable (major dissection: 0.86 ± 0.05, fracture: 0.84 ± 0.06, neither: 0.86 ± 0.05; *p* = 0.053). Multilevel logistic regression, adjusted for individual patients and OFDI parameters, found both dissection (OR: 1.70, 95% CI 1.17–2.47, *p* = 0.01) and fracture (OR: 1.57, 95% CI 1.01–2.44, *p* = 0.05) independently associated with optimal lumen expansion after stenting.

This study suggests that major dissections, like fractures, play a key role in IVL by modifying calcified plaques and facilitating stent expansion. More specifically, the presence of major dissections on OFDI following IVL may serve as an important predictor of optimal lumen expansion, even in the absence of visible fractures. Further studies with larger sample sizes are warranted to clarify the clinical significance of major dissections observed on OFDI after IVL therapy.

## Data Availability

The study protocol prohibits open access to the data. Nevertheless, interested parties may request additional analyses from the corresponding author, and we will accommodate such requests to the best of our ability.
