# Correction: The potential role of major dissection observed on optical frequency-domain imaging in optimal lumen expansion after coronary intravascular lithotripsy: a comparative analysis with calcium fractures

**DOI:** 10.1007/s12928-025-01167-w

**Published:** 2025-07-21

**Authors:** Kotaro Miyata, Taku Asano, Takahiro Suzuki, Masafumi Ono, Jiro Aoki

**Affiliations:** Department of Cardiovascular Medicine, St. Luke’s International Hospital, St. Luke’s International University, 9-1 Akashi-Cho, P. O. Box 104-8560, Chuo-Ku, Tokyo Japan

**Correction: Cardiovascular Intervention and Therapeutics** 10.1007/s12928-025-01145-2

The article “The potential role of major dissection observed on optical frequency-domain imaging in optimal lumen expansion after coronary intravascular lithotripsy: a comparative analysis with calcium fractures”, written by Kotaro Miyata, Taku Asano, Takahiro Suzuki, Masafumi Ono and Jiro Aoki, was originally published under exclusive license to The Author(s) under exclusive licence to Japanese Association of Cardiovascular Intervention and Therapeutics. As a result of the subsequent decision to publish the article under the open access model, the article’s copyright notice was changed on 2 July 2025 to © The Author(s) 2025 and the article is now distributed under a Creative Commons Attribution 4.0 International License, which permits use, sharing, adaptation, distribution and reproduction in any medium or format, as long as you give appropriate credit to the original author(s) and the source, provide a link to the Creative Commons licence, and indicate if changes were made. The images or other third party material in this article are included in the article’s Creative Commons licence, unless indicated otherwise in a credit line to the material. If material is not included in the article’s Creative Commons licence and your intended use is not permitted by statutory regulation or exceeds the permitted use, you will need to obtain permission directly from the copyright holder. To view a copy of this licence, visit http://creativecommons.org/licenses/by/4.0/.

The original article has been corrected.

